# Correlations among Antibiotic Resistance Genes, Mobile Genetic Elements and Microbial Communities in Municipal Sewage Treatment Plants Revealed by High-Throughput Sequencing

**DOI:** 10.3390/ijerph20043593

**Published:** 2023-02-17

**Authors:** Fuzheng Zhao, Bo Wang, Kailong Huang, Jinbao Yin, Xuechang Ren, Zhu Wang, Xu-Xiang Zhang

**Affiliations:** 1 Key Laboratory of Yellow River Water Environment in Gansu Province, Lanzhou Jiaotong University, Lanzhou 730070, China; 2 School of Environmental and Municipal Engineering, Lanzhou Jiaotong University, Lanzhou 730070, China; 3 State Key Laboratory of Pollution Control and Resource Reuse, School of the Environment, Nanjing University, Nanjing 210023, China; 4 Institute of Environmental Research at Greater Bay, Key Laboratory for Water Quality and Conservation of the Pearl River Delta, Ministry of Education, Guangzhou University, Guangzhou 510006, China

**Keywords:** antibiotic resistance genes, mobile genetic elements, bacterial community structure, high-throughput sequencing, metagenomic, sewage treatment plant

## Abstract

Municipal sewage treatment plants (MSTPs) are environmental pools for antibiotic resistant bacteria (ARB) and antibiotic resistance genes (ARGs), which is cause for growing environmental-health concerns. In this study, the effects of different wastewater treatment processes on microbial antibiotic resistance in four MSTPs were investigated. PCR, q-PCR, and molecular cloning integrally indicated that the tetracycline resistance (*tet*) genes significantly reduced after activated-sludge treatment. Illumina high-throughput sequencing revealed that the broad-spectrum profile of ARGs and mobile element genes (MGEs) were also greatly decreased by one order of magnitude via activated sludge treatment and were closely associated with each other. Correlations between ARGs and bacterial communities showed that potential ARB, such as *Acinetobacter*, *Bacteroides*, and *Cloaibacterium*, were removed by the activated-sludge process. Sedimentation processes cannot significantly affect the bacterial structure, resulting in the relative abundance of ARGs, MGEs, and ARB in second-clarifier effluent water being similar to activated sludge. A comprehensive study of ARGs associated with MGEs and bacterial structure might be technologically guided for activated sludge design and operation in the MSTPs, to purposefully control ARGs carried by pathogenic hosts and mobility.

## 1. Introduction

Overuse and misuse of antibiotics in the health protection of humans and animals have resulted in the emergence and spread of various antibiotic resistance genes (ARGs) and antibiotic-resistant bacteria (ARB) in the environment [1], which has brought enormous challenges including threats to environmental safety [2] and human health [3].

Municipal sewage treatment plants (MSTPs) serve as important reservoirs of environmental ARB and ARGs [4]. MSTP sewage receives gut bacteria from households, hospitals, and animal husbandry previously exposed to antibiotics, which was the dominant force shaping the bacterial profile of influent sewage [5]. Some species of bacteria were confirmed ARB, such as *Acinetobacter* spp. [6], *Bacteroides* spp. [7] and *Cloacibacterium* spp. [8]. In these cases, bacteria can capture different ARGs through horizontal gene transfer (HGT) by mobile genetic elements (MGEs), including plasmids, integrons, and insertion sequences (ISs) [9], given high biomass and various selective pressures, so that MGEs can promote the dissemination of ARB and ARGs in the environment [10]. The mobile resistome can easily spread among bacterial species including human pathogens, which deserves greater public health concern [11]. Previous studies have suggested that the activated-sludge process can reduce the levels of various ARGs in the given volume of sewage by decreasing microbial biomass [12,13].

Culture-based methods [14], and molecular methods including PCR [15], molecular cloning [16], and microarray [17], are often used to detect environmental ARB and ARGs, but the full investigation of ARGs is still limited. Recently, high-throughput sequencing-based metagenomic analysis has been considered a promising culture-independent method to determine the diversity and abundance of ARGs in the environment [18,19,20], which has also shown great advantages in microbial-community profiling due to its unprecedented sequencing depth [11,18,21]. MGEs can contribute to the HGT of ARGs among different microbial organisms, and were considered as an indicator of the HGT potential and multiple antibiotic resistance [9,22,23]. In addition, ARGs can interact with the diverse bacterial communities in MSTPs. For instance, two similar bacterial communities contained a co-occurrence genus, in which the co-occurrence genus is ARB in one community but is not ARB in another, suggesting that the co-occurrence genus have the same habitats, but have different sensitivities and tolerances to specific antibiotics, so that ARGs can affect bacterial communities under correlational-antibiotic selective pressures. Conversely, two different communities contained the same ARGs, in which each ARG was contained in the different bacteria from two different communities, indicating that ARB have different suitabilities under specific environmental conditions, and that bacterial communities can affect ARGs under those conditions. Hence, the correlation among ARGs, MEGs, and bacterial communities can be assessed to explore a comprehensive resistome in MSTPs.

In this study, metagenomic analyses based on Illumina high-throughput sequencing and 454 pyrosequencing were used to comprehensively investigate the abundance and diversity of ARGs, MGEs, also bacterial species and their correlations in MSTPs. This study might be practically useful for the detection of ARGs in water environments and assessment of their environmental fates, and the results may help to extend our knowledge of antibiotic resistance in MSTPs.

## 2. Materials and Methods

### 2.1. MSTPs and Samples Collection

Water samples were collected from four MSTPs geographically located in three cities in China. Table S1 shows detailed information about the MSTPs. Sewage in the four MSTPs undergoes different treatment processes (Table S1). Influent water (IW), activated sludge (AS), and effluent water (EW) of the aerobic tanks were sampled from each MSTP at three or six time points (Table S1). Water and sludge samples were cooled in an ice bath and transported to the lab for immediate processing.

The IW (200 mL) and EW (1200 mL) were concentrated by filtration onto a cellulose ester filter (0.45-µm-pore-size), and the AS (6 mL) samples were centrifuged at 14,000× *g* for 1 min at 4 °C before the supernatant was discarded. Subsequently, the filtered and centrifuged materials were used for total genomic DNA extraction using the FastDNA Soil Kit (MP Biomedicals, CA, USA). The DNA purity and concentration were detected by microspectrophotometry (NanoDrop^®^ND-2000, NanoDrop Technologies, Wilmington, DE, USA).

### 2.2. PCR of Tetracycline Resistance Genes

Polymerase chain reactions (PCRs) were conducted to investigate the occurrence of 15 tetracycline resistance genes (*tet*) in JXZ-MSTP, including nine efflux protein genes (*tet*A, *tet*B, *tet*C, *tet*D, *tet*E, *tet*G, *tet*K, *tet*L, and *tet*A(P)), five ribosomal protection proteins genes (*tet*M, *tet*O, *tet*Q, *tet*S, and *tet*W), and one oxidoreductase gene (*tet*X). The reaction systems (30 µL each) contained 1 × PCR buffer, 100 µM dNTP, 2 µM of each primer set (Table S2), 50 ng of template DNA, and 1 U of ^EX^Taq polymerase (TaKaRa, Japan). PCR conditions were classified into three groups for the different *tet* genes as follows: (1) denaturation at 94 °C for 5 min, followed by 35 cycles of 1 min at 94 °C, annealing at 55 °C (*tet*A, *tet*C, *tet*K, *tet*M, *tet*A(P) and *tet*X), 55.5 °C (*tet*G), 56 °C (*tet*B, *tet*D, and *tet*L), 56.5 °C (*tet*S) or 57 °C (*tet*E) for 1 min and extension at 72 °C for 1 min, and a final extension at 72 °C for 7 min; (2) denaturation at 94 °C for 5min, followed by 30 cycles of 30 s at 94 °C, annealing at 58 °C (*tet*Q) or 64 °C (*tet*W) for 30 s and extension at 72 °C for 30 s, and a final extension at 72 °C for 7 min; (3) denaturation at 95 °C for 7 min, followed by 40 cycles of 15 s at 94 °C, annealing at 50.3 °C (*tet*O) for 30 s and extension at 72 °C for 30 s, and a final extension at 72 °C for 7 min. PCR products were analyzed by gel electrophoresis using 1% (*w*/*v*) agarose in 1 × TAE buffer. To guarantee reproducibility, duplicate PCR reactions were performed for each sample. Sterile water and plasmids containing the target gene were used as the negative control and positive control for each assay, respectively.

### 2.3. Cloning and Phylogenetic Analysis of Tet Genes

*Tet*G was selected for assessment of its gene diversity in IW, AS, and EW in JXZ-MSTP using molecular cloning. The PCR products of *tet*G generated from different samples were individually purified using a DNA Fragment Purifiction Kit (TaKaRa Bio Inc., Shiga, Japan), connected with pMD18-T Vector (TaKaR, Japan) and finally transformed to JM109 competent cells. After positive recombinants were identified with PCR using M13F/M13R primer set, colonies (45 ones for IW, 42 clones for AS, and 45 clones for EW) carrying recombinant plasmids were randomly selected for library construction. The plasmids were sequenced by using M13F and M13R primers on ABI 3730xl capillary sequencers (PE Applied Biosystems, Foster City, CA, USA). Operational taxonomic units (OTUs) were generated from the sequences using Mothur software (https://github.com/mothur/mothur/releases) [24]. The representative sequence of each OUT was compared against the National Center for Biotechnology Information (NCBI) Nucleotide Database using the BLASTn algorithm to obtain the reference sequences from the NCBI GenBank (https://ftp.ncbi.nih.gov/genbank/). The neighbor-joining phylogenetic trees were constructed using MEGA 5.0 software (https://www.megasoftware.net/) via bootstrap analysis of 1000 replicates. The 132 DNA sequences of *tet*G obtained in this study have been deposited in GenBank under accession number 1648995.

### 2.4. q-PCRs of Tet Genes

Eight *tet* genes including *tet*C, *tet*E, *tet*G, *tet*M, *tet*O, *tet*Q, *tet*W, and *tet*X were selected for quantitative real time polymerase chain reaction (q-PCR) analysis to determine the abundance of the ARGs in the monthly samples of IW, AS, and EW of JXZ-MSTP. To determine the *tet* abundance, the plasmids containing target genes were obtained by molecular cloning to generate standard curves for the q-PCRs. Five- to six-point calibration curves (Ct values versus the log of initial target gene copy) were generated using a ten-fold serial dilution of the plasmid carrying each *tet* gene [13]. To avoid potential variations in DNA extraction efficiencies, eubacterial 16S rRNA gene sequences were used as housekeeping genes to normalize the *tet* gene abundance, following Lopez-Gutierrez et al. (2004). The reaction systems (20 µL each) contained 10 µL of SYBR Premix ^EX^Taq Super Mix (TaKaRa, Japan), 0.2 µL of each primer (10 µM) (Table S2), 1.6 µL of ddH_2_O, and 8 µL of DNA templates (1 ng/µL) [13]. Thermal cycling and fluorescence detection were conducted on Corbett Real-Time PCR Machine with the Rotor-Gene 6000 Series Software (version 1.7, QIAGEN, Venlo, The Netherlands) using the following protocol: denaturation at 94 °C for 3min, followed by 40 cycles: (1) denaturation at 94 °C for 30 s, annealing at 58 °C (*tet*C, *tet*O, and *tet*Q) for 45 s, and extension at 72 °C for 45 s; (2) denaturation at 94 °C for 15 s, annealing at 56 °C (*tet*M) for 15 s, and extension at 72 °C for 30 s; (3) denaturation at 94 °C for 10 s, annealing at 56 °C (*tet*W) for 10 s, and extension at 72 °C for 15 s; (4) denaturation at 94 °C for 15 s, annealing at 56 °C (*tet*E, *tet*M, and *tet*X) or 58 °C (*tet*G) for 15 s, and extension at 72 °C for 20 s; (5) denaturation at 94 °C for 20 s, annealing at 56 °C (16S rRNA gene) for 40 s, and extension at 72 °C for 40 s. Each reaction was run in triplicate for each sample. PCR efficiency of the *tet* gene ranged from 80.0% to 100.5%, with the R^2^ value being over 0.993 for the calibration curves. The specificity of the qPCRs was confirmed by observation of melting-curve and gel-electrophoresis of the products.

### 2.5. High-Throughput Sequencing

An equal mass of each DNA sample from the sewage or sludge sampling at different time points was pooled together to minimize the temporal variation. The environmental metagenomes were sent out to Jiangsu Zhongyijinda Analytical and Testing Co., Ltd. (Yixing, Jiangsu, China) for shotgun-library construction and Illumina high-throughput sequencing using the HiSeq 2000 platform (Illimina, San Diego, CA, USA). The sequencing strategy for Index 101 PE (Paired-End sequencing, 101-bp reads, and 8-bp index sequence) was applied to generate a nearly-equal amount of sequencing reads. Quality control and annotation of ARGs and MGEs were described in the previous study [19].

The hypervariable V3–V4 region of the 16S rRNA gene was amplified from the pooled DNA samples of IW, AS, and EW from JXZ-MSTP and DC-MSTP, following Claesson et al. [25]. The sequences of the forward and reverse primers were V3F (5’- ACTCCTACGGGAGGCAGCAG-3’) and V4R (5’- TACNVGGGTATCTAATCC-3’), respectively. The 10 nucleotide “barcodes” were assigned to each sample to distinguish the consistent reads from the data pool generated in a single pyrosequencing run [26]. Each PCR reaction solution (50 μL) contained 1 × Pfx Amplification Buffer (Invitrogen, Waltham, MA, USA), 0.4 mM dNTP, 2 mM MgSO4, 0.4 μM each primer, 1 μL of template DNA, and 2 U of Platinum^®^ Pfx DNA Polymerase (Invitrogen, USA). The PCR amplification was conducted according to the following conditions: initial denaturation at 94 °C for 3 min, followed by 30 cycles of 94 °C for 30 s, annealing at 62 °C for 30 s, and extension at 70 °C for 45 s, with a final elongation step at 70 °C for 7 min. The purified PCR products were qualified using Agilent 2100 Bioanalyzer (Agilent, Santa Clara, CA, USA) and then mixed accordingly to achieve an equal DNA mass in the final mixture for each sample, which was sent out to Beijing Genome Institute (Shenzhen, China) for pyrosequencing using the Roche 454 FLX Titanium platform (Roche, Branford, CT, USA). Analysis of Taxonomy and diversity was described in the previous study [27].

### 2.6. Statistics

Linear regression analysis of the abundance of the total ARGs and MGEs was carried out by using the Statistical Product and Service Solutions software (SPSS, version 21.0). Additionally, Canonical Correspondence Analysis (CCA) was conducted to investigate the relationship between the bacterial communities and the ARGs abundance of the IW, AS, and EW in the four MSTPs using the “vegan” package (version 2.0-9) of the R software (version 3.0.2). Cluster Analysis (CA) was also carried out based on the Pearson correlation matrix of the abundance of the ARGs and bacteria, integrase genes, and insertion sequences in the IW, AS, and EW of the MSTPs by using PAleontological STatistics software (PAST, version 3.01).

## 3. Results

### 3.1. Occurrence and Abundance of Tet Genes in JXZ-MSTP

**Figure 1:**
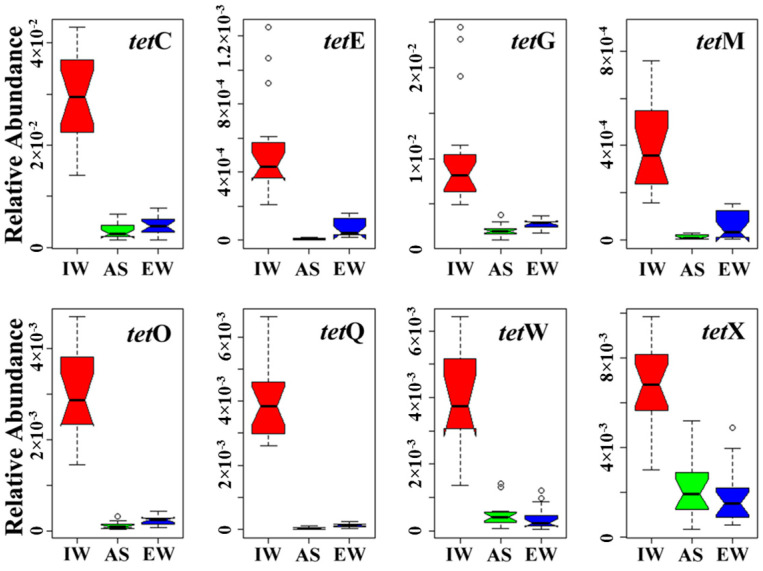
Total abundance of 8 tetracycline-resistance genes in influent water (IW, red), activated sludge (AS, green), and effluent water (EW, blue), collected from Jiangxinzhou municipal sewage treatment plants (JXZ-MSTP). The abundance was normalized to the total 16S rRNA genes. If the notches on each side of the boxes of the two plots do not overlap, then the medians are significantly different at the 5% level, which suggests that the median concentrations at the three sites as shown are statistically different from each other (o: mild outlier).

A traditional molecular biology method was performed to focus on long-term monitoring of important ARGs (*tet* genes) in JXZ-MSTP. PCRs showed that the 15 *tet* genes tested in this study occurred in JXZ-MSTP (Figure S1). Among the eight genes quantified, the rangeability was relatively small over six months, and *tet*C had the most copies in each sample (*p* < 0.05), followed by *tet*G and *tet*X (Figure S2). After being normalized to 16S rRNA, each gene had significantly higher abundance in IW than in AS and EW (*p* < 0.05), and *tet*E, *tet*G, *tet*M, *tet*O, and *tet*Q seemed to have higher abundance in EW than in AS (*p* < 0.05) (Figure 1). Interestingly, the relative abundance of *tet*E, *tet*Q, *tet*M, and *tet*O (normalized to 16S rRNA gene) was reduced by over 90% after the sewage entered into the aerobic tank but was then increased by over two-fold in the treated sewage. Phylogenic analysis of *tetG* gene showed that the eight OTUs generated from the 132 *tet*G clones of three samples were mainly grouped into two types (Figure S3). The two OUTs from IW were aligned to one group affiliated with the gene *tetG* carried by *Ochrobactrum* sp. The other six OTUs from AS and EW were closely grouped.

### 3.2. Abundance and Diversity of ARGs in Four MSTPs

**Figure 2:**
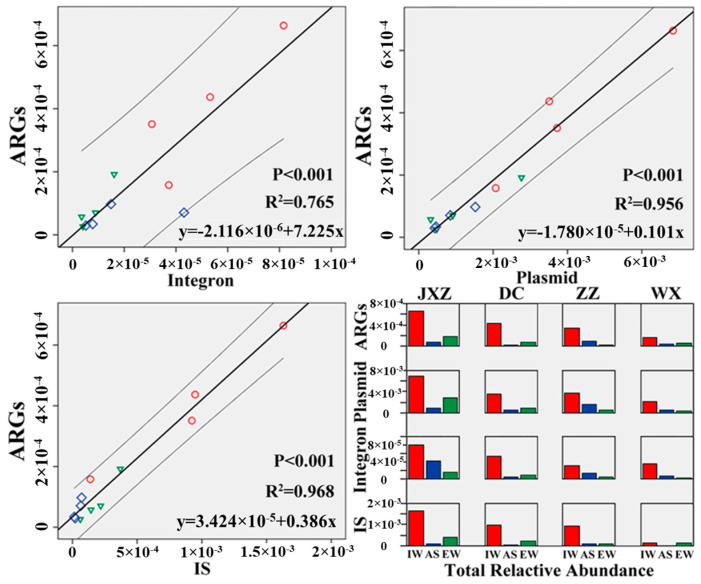
The total relative abundance and correlations between the relative abundance of ARGs and MGEs (plasmid, integrase genes, and insertion sequence) for samples including influent water (IW), activated sludge (AS), and effluent water (EW) in four municipal sewage treatment plants (MSTPs). (Red Circle: IW; Blue Rhombus: AS; Green Triangle: EW).

**Figure 3:**
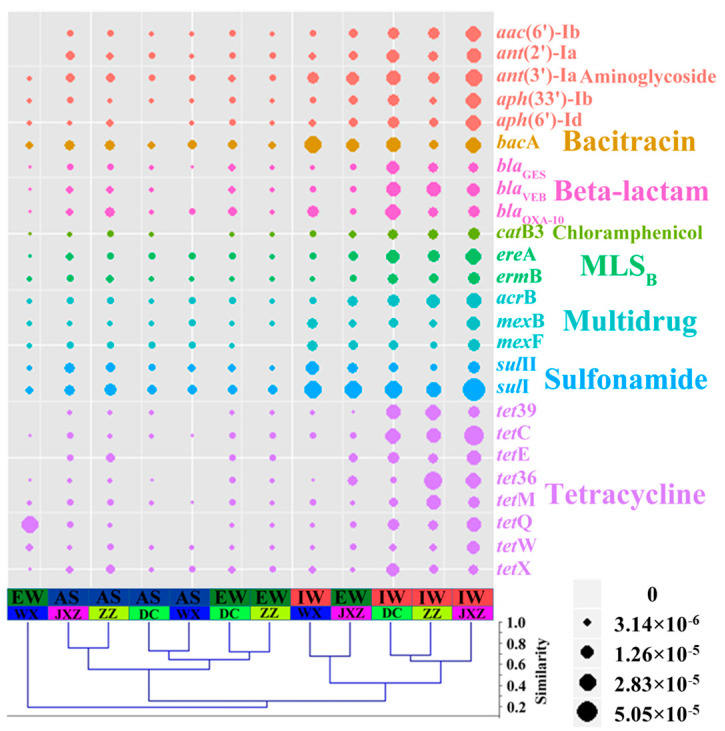
The distributions of ARGs (>1 × 10^−5^ relative abundance at least one sample) in samples including influent water (IW), activated sludge (AS), and effluent water (EW) in four municipal sewage treatment plants (MSTPs). The Cluster Analysis (CA) is based on a distance matrix computed using Bray–Curtis similarity of 12 samples.

Illumina high-throughput sequencing was then used to comprehensively investigate the broad-spectrum profile of ARGs in the four MSTPs. BLAST against the ARDB showed that the relative abundance of all ARGs in IW was higher than those of AS and EW in each MSTP (Figure 2, bottom right panel). After the activated-sludge process, over 70% of the ARGs detected in IW were eliminated (JXZ-MSTP: 89.37%, DC-MSTP: 93.25%, WX-MSTP: 78.39%, ZZ-MSTP: 72.19%). Similar to q-PCR, the metagenomic analysis showed that the relative abundance of the ARGs was reduced after the sewage entered the aerobic tanks (Figure 3). Compared with AS, IW had a higher proportion of most of the ARGs, especially the genes encoding tetracycline resistance (Figure S4). Among the detected ARGs, the dihydropteroate synthase gene *sul*I that confers resistance to sulfonamides had a higher relative abundance (>1 × 10^−6^) in each sample from the MSTPs (Figure 3). Cluster analysis showed that AS and EW usually had similar distribution patterns of ARGs, each of which was comparatively different from IW (Figure 3).

### 3.3. Correlation between ARGs and MGEs in the MSTPs

To explore the correlation between ARGs and MGEs in the MSTPs, we also searched the metagenomes for plasmids, integrons, and insertion sequences. Alignment against the INTEGRALL database, NCBI RefSeq database, and IS Finder database revealed the occurrence of a variety of integrase genes, plasmids, and ISs in the MSTPs (Figure 2, bottom right panel). IW had a higher abundance of integrase genes, plasmids, and ISs than AS and EW (Figure 2, bottom right panel). For instance, the total abundance of plasmids in IW was generally over two-fold greater than those in AS (2.4 fold for ZZ-MSTP, 4.5 fold for WX-MSTP, and about eight-fold for JXZ-MSTP and DC-MSTP) (Figure 2, bottom right panel).

Statistical analysis showed that the total abundance of ARGs had a significant correlation with the total abundance of each MGE (integrase genes, plasmids, and ISs) in the MSTPs (*p* < 0.001 each) (Figure 2, top left panel, top right panel, and bottom left panel). Among the integrases genes, *intI1* had the highest abundance in the MSTPs (Figure S5) and was found to be closely related to the abundance of 18 ARGs, encoding resistance to tetracyclines (*tetC*, *tetW*, *tet*39), sulfonamide (*sul*I, *sul*II), aminoglycoside (*ant*(2’)-Ia, *ant*(3’)-Ia, *aph*(6’)-Id, *aph*(33’)-Ib, *aac*(6’)-Ib), beta-lactam (*bla*_GES_, *bla*_VEB_), chloramphenicol (*cat*B3), MLS_B_ (*ere*A, *erm*B), and multidrug (*acr*B, *mex*B, *mex*F) (Figure S6). Additionally, a significantly positive correlation was found between the quantities of *intI2* and 13 ARGs (R > 0.5; *p* < 0.05), but other types of integrons showed notable correlations with fewer ARGs (Figure S6). Among the 25 ISs analyzed in this study, 23 ones were found to quantitatively correlate with one or more ARGs, and the abundance of each tested ARG had a positive correlation with the quantity of at least one IS (Figure S7). IS*Aba1* had the highest abundance than other ISs in the MSTPs (Figure S8), which showed a positive correlation with the abundance of various ARGs encoding to tetracyclines (*tet*C, *tet*E, *tet*W, *tet*36, *tet*39), sulfonamide (*sul*I, *sul*II), aminoglycoside (*ant*(2’)-Ia, *ant*(3’)-Ia, *aph*(6’)-Id, *aph*(33’)-Ib, *aac*(6’)-Ib), beta-lactam (*bla*_GES_, *bla*_VEB_), chloramphenicol (*cat*B3), MLSB (*ere*A, *erm*B), multidrug (*acr*B, *mex*B, *mex*F) (Figure S7).

### 3.4. Correlation between ARGs and Bacterial Communities in MSTPs

**Figure 4:**
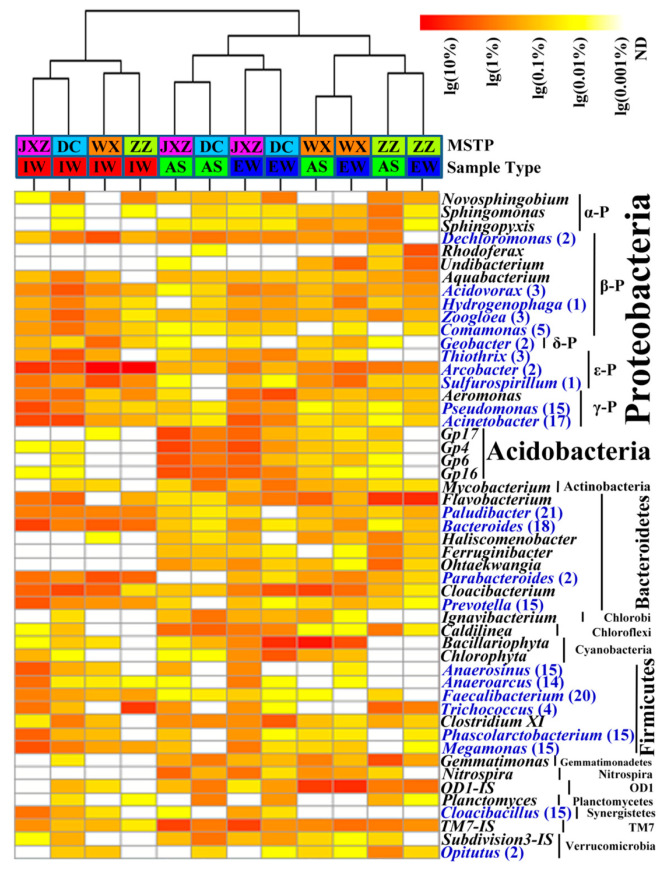
Heatmap illustrating the relative abundance of genera occurring at >1% in at least one sample. The scale bar shows the variation range of the normalized abundance of the genera. The ‘blue’ genus was defined as “potential antibiotic resistant bacteria” and the tail number represented the number of correlational ARGs shown in Figure 6.

**Figure 5:**
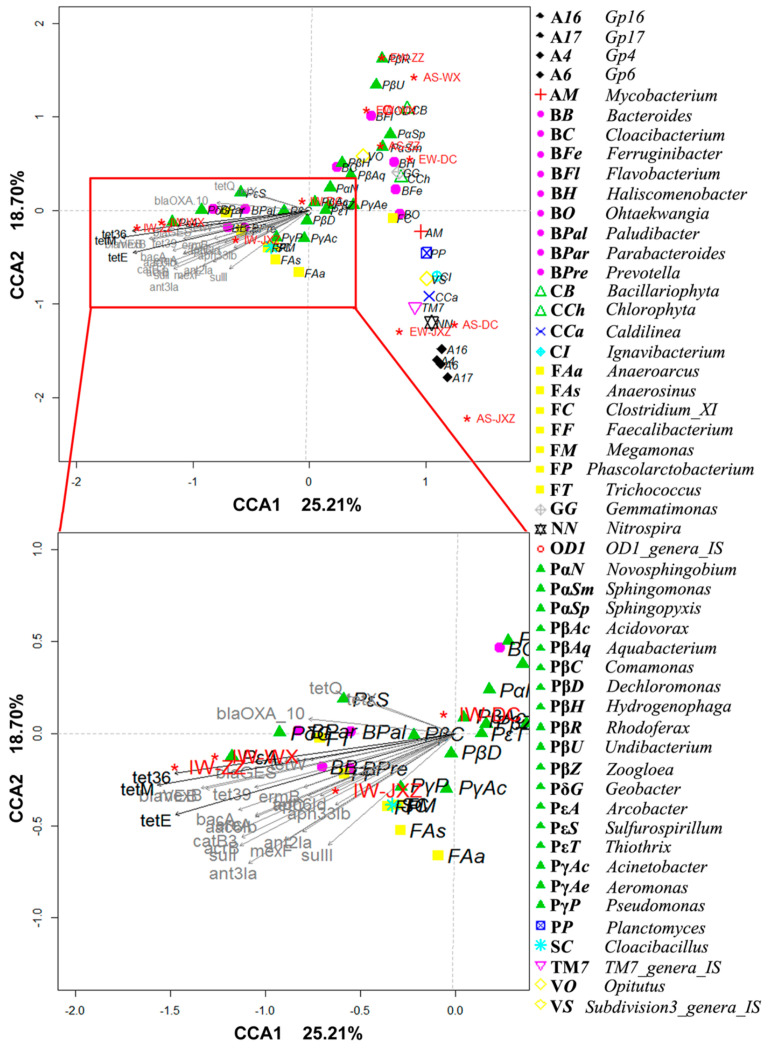
Correspondence Canonical Analysis (CCA) of the relative abundance of the genus (>1%) in the function of the relative abundance of antibiotic resistance genes (>1 × 10^−5^ relative abundance in at least one sample). The percentage of variation explained by each axis is shown. The different shapes of points represent different phyla, which were sequentially: Acidobacteria, Actinobacteria, Bacteroidetes, Cyanobacteria, Chloroflexi, Chlorobi, Firmicutes, Gemmatimonadetes, Nitrospira, OD1, Proteobacteria, Planctomycetes, Synergistetes, TM7, and Verrucomicrobia. Red star represented WWTPs samples. (Black arrows: *p* < 0.1 (*tet*36: *p* = 0.095; *tet*E: *p* = 0.049; *tet*M: *p* = 0.048); gray arrows: *p* > 0.1) (*p* values based on 2000 permutations in *envfit* function, vegan, and permute package of R).

Pyrosequencing was used to characterize the microbial structure shift along the treatment processes in the MSTPs. Cluster analysis showed that the microbial communities of AS and EW were grouped at the genus level, and diverged from those of IW (Figure 4). The correlation was examined through CCA, based on the abundance of the 51 genera (>1%) and the abundance of the 25 identified ARGs (>1 × 10^−5^) in the samples collected from four MSTPs. ARGs were analyzed as the environmental factors for genera. As shown in Figure 5, among the 25 identified ARGs, especially *tet*E and *tet*M, were significantly correlated with the distribution of the genus in MSTPs (*p* < 0.05), indicating that these ARGs possibly played important roles in shaping the genus distribution in MSTPs. The pivotal ARGs were divided into two groups: one group belonging to the phyla Firmicutes, Bacteroidetes, Proteobacteria, and Synergistetes dominant in IW, and the other group dominant in AS and EW of the MSTPs. In other words, ARGs encoding resistance to tetracycline antibiotics were positively correlated with the genera dominating in IW, while also having negative correlations with the genera dominating in AS (Figure 5).

**Figure 6:**
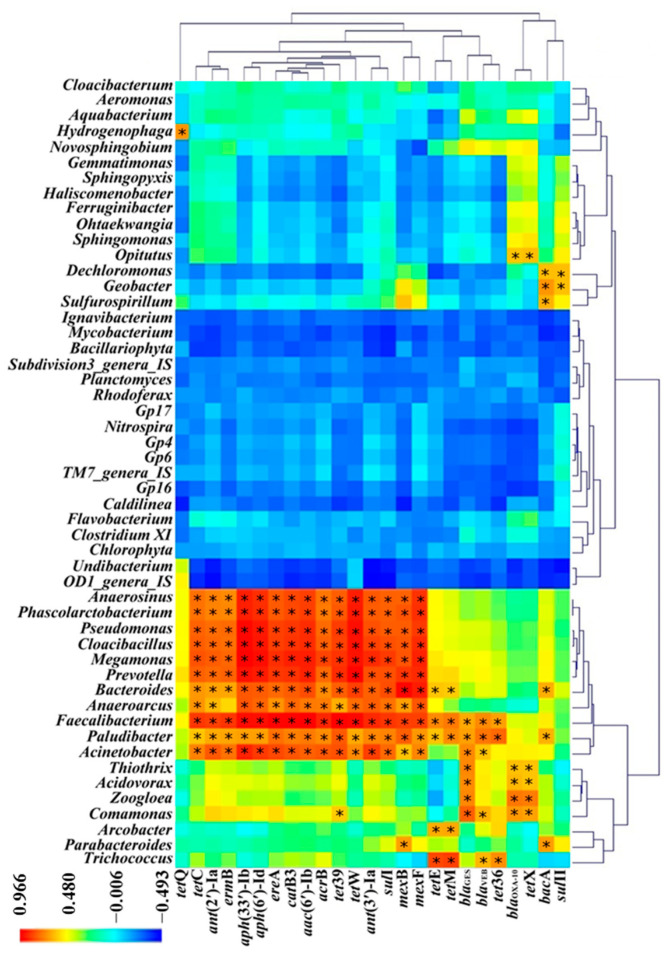
Heatmap illustrating the Pearson correlation coefficients among genera occurring at >1% relative abundance in at least one sample, and ARGs (>1 × 10^−5^ relative abundance in at least one sample) in samples including influent water (IW), activated sludge (AS), and effluent water (EW) in four municipal sewage treatment plants (MSTPs) (*n* = 12). The Cluster Analysis (CA) is based on a distance matrix computed with Bray–Curtis similarity of the 25 ARGs and 51 genera. The scale bar shows the variation range of the correlation coefficient. (*: R > 0.5; *p* < 0.05).

To explore the correlation between each ARG and each genus, the Pearson correlation coefficient revealed that the abundance of each ARG was significantly positively correlated with at least one genus (R > 0.5; *p* < 0.05) (Figure 6). In particular, the abundance of a group of ARGs including tetracyclines (*tet*E, *tet*W, *tet*39), sulfonamide (*sul*I), aminoglycoside (*ant*(2’)-Ia, *ant*(3’)-Ia, *aph*(6’)-Id, *aph*(33’)-Ib, *aac*(6’)-Ib), chloramphenicol (*cat*B3), MLS_B_ (*ere*A, *erm*B), multidrug (*acr*B, *mex*B, *mex*F), was all correlated with several genera including Firmicutes (*Anaerosinus*, *Phascolarctobacterium*, *Megamonas*, *Anaeroarcus,* and *Faecalibacterium*), Proteobacteria (*Pseudomonas* and *Acinetobacter*), Synergistetes (*Cloacibacillus),* and Bacteroidetes (*Prevotella*, *Bacteroides* and *Paludibacter*), except for the correlation between *erm*B and *Anaeroarcus*.

ARDB is a special database about antibiotic resistance, in which each gene and resistance type is annotated with rich information, especially including resistance profile, mechanism of action, and original host. In ARDB, the 41 subtypes ARGs entire sequences were found in *Acinetobacter*, including *tet*C (1 sequence of entire *tet*C gene), *aph*(6’)-Id (3), c*at*B3 (10), *aac*(6’)-Ib (1), *tet*39 (1), *sul*I (11), *tet*M (1), *bla*_GES_ (1), *bla*_VEB_ (4), *bla*_OXA-10_ (4) and the 13 subtypes ARGs entire sequences was found in *Bacteroides*, including *tet*Q (15), *tet*C (1), *tet*36 (1), *tet*X (2) related to this study (Table S3).

Among the potential ARB, the relative abundance of 15 potential ARB was more than 1% in IW, especially for the *Arcobacter* genus (average accounting for 33.82%), *Bacteroides* genus (3.45%), and *Acinetobacter* genus (3.24%) in Figure 4, detected as the most resistant genus in the IW. After the activated-sludge process, the relative abundance of potential ARB was generally decreased to fewer than 33%, except for lower potential ARB correlated with a few ARGs, including *Acidovorax*, *Hydrogenophaga*, *Dechloromonas,* and *Zoogloea*.

## 4. Discussion

In this study, PCRs and q-PCRs showed that the different types of *tet* genes prevailed in JXZ-MSTP. Tetracycline is one of the most commonly-used therapeutics in human and veterinary medicine [28], and is present in sewage [28] and activated sludge [16]. Recently, several previous studies have indicated that *tet* genes occur in MSTPs with high abundance [12]. For a comprehensive investigation of ARGs in MSTPs, this study used metagenomic analysis to reveal that a variety of ARGs encoding resistance to various antibiotics, including tetracycline, sulfonamide, aminoglycoside, β-lactam, chloramphenicol, trimethoprim and vancomycin, was present in the raw and treated sewage, which agrees with previous studies indicating that sewage treatment plants serve as important environmental pools of ARGs [29]. Among the detected ARGs, *sul*I had the highest relative abundance in the MSTP. This result is supported by Yang et al.’s study indicating that the sulfonamide resistance genes are often prevalent in MSTPs [20].

Metagenomic analysis and q-PCR applied in this study consistently indicated that the conventional sewage treatment processes could effectively decrease the relative abundance of various ARGs normalized to the total microbial community in sewage. Previous studies have also indicated that the activated sludge process can reduce the levels of various ARGs in a given volume of sewage by decreasing microbial biomass [12,13]. Two reasons may explain the dilution of ARGs in the sewage after entering the treatment system. Firstly, many antibiotics may be removed in the activated-sludge process by biodegradation and/or adsorption [30]. Secondly, the microbial structure shift from IW to AS and EW may contribute to the decrease of ARGs abundance in the sewage. The possible explanation is that the growth of the ARB dominant in anaerobic or anoxic environments of IW was inhibited when the sewage entered aerobic tanks.

To test the hypothesis that the bacterial communities may affect the abundance and diversity of the ARGs in MSTPs, we used pyrosequencing to investigate the microbial community structure in the IW, AS, and EW. Similarly to Shanks et al.’s study [31], this study showed that the phyla Proteobacteria, Firmicutes, and Bacteroidetes dominated the raw sewage. It is known that the anaerobic members within the phyla Bacteroidetes and Firmicutes numerically and functionally dominate the intestinal microbiota of mammals [18,21], so they are often used as indicators for the detection and characterization of fecal pollution [32]. This study showed that the phyla had comparatively lower abundance in AS, which is supported by Sanapareddy et al.’s study [33] demonstrating that phylotypes related to non-fecal sources numerically dominate human fecal phylotypes in AS. The prevalence of the phyla may account for the high abundance of ARGs in the IW samples, since Bacteroidetes are the common bacteria resistant to various antibiotics including fluoroquinolone [34]. Firmicutes can confer resistance to antimicrobial peptides, and were often found to carry ATP-binding cassette transporters recognized as important resistance determinants, with relatively higher detection frequencies than other bacteria [35].

Correlation analysis showed that the abundance of some genera, including *Acinetobacter*, *Bacteroides*, *Cloacibacterium*, *Paludibacter*, *Parabacteroides* and *Faecalibacterium*, had significantly positive correlations with the copies of ARGs in the MSTPs. The genera dominated in the IW, but many of them had comparatively lower abundance in the AS. *Acinetobacter* prevalent in the IW was found to have an extremely high abundance in the human gut [35]. Vila et al.’s study [36] revealed the location of various ARGs and integrons in *Acinetobacter* strains isolated from human feces. In Salyers et al.’s study [7], *Bacteroides* dominating the human gut usually harbored two types of conjugative elements: plasmids and transposons, as well as various ARGs including *tet*Q and *erm*F. Although the relationship between *Bacteriodes* and *tet*Q/*erm*F was not found in this study, some types of ARGs, such as *tet*39, *tet*C, and *tet*W with the same type of *tet*Q, and *erm*B with the same type of *erm*F, have a significantly positive correlation with *Bacteriodes* confirmed in correlation analysis. A search in ARDB also showed that most species of *Acinetobacter* and *Bacteroides* carry the complete structure of a large number of ARGs. Allen et al. (2006) indicated that *Cloacibacterium* isolates from MSTPs were resistant to streptomycin, erythromycin, nalidixic acid and kanamycin.

To determine the MGEs responsible for HGT of ARGs in MSTPs, we used high-throughput sequencing and metagenomic analysis to quantify plasmids, ISs, and integrons. This study showed that the MGEs detected in the IW were diluted after aerobic treatment of the sewage, which agrees with Ma et al.’s study [37], demonstrating a decreasing trend of MGE abundance along sewage treatment processes. Furthermore, this study revealed numerous positive correlations between some ARGs and MGEs (integrons and ISs). Among the different types of integrase genes, *int*I1 had the highest abundance in the MSTPs and was correlated with various ARGs. A previous study showed that the abundance of *sul*I had a significantly positive correlation with the quantity of *int*I1 [37]. *Sul*I is known as a part of the conserved region on class 1 integrons [38]. Previous studies have shown that ARGs coding for resistance to various antibiotics, e.g., aminoglycosides [34] and β-lactams [29], are often included in gene cassettes carried by class 1 integrons [39]. These integron-gene cassette systems associated with multiple resistance were frequently isolated from the bacterial species dominating the MSTPs, e.g., *Acinetobacter baumannii* [22]. Most of the ISs detected in this study were found to quantitatively correlate with at least one ARG. IS*Aba1* had the highest abundance in the IW, and the genus of *Acinetobacter* containing at least 13 copies of IS*Aba1* can confer resistance to all clinically useful antibiotics [23].

## 5. Conclusions

The combination of traditional molecular biological technology and metagenomics reveals the prevalence of a variety of ARGs in MSTPs, and correlations of ARGs with MGEs and bacterial communities in sludge-treatment water plants. This correlation analysis method, based on high-throughput sequencing and metagenomic analysis, can also be used to conduct the broad-spectrum scan of different potential antibiotic-resistant bacteria for other types of samples including water, sediments, soils, and feces to facilitate the subsequent molecular biology experiment to confirm antibiotic-resistant bacteria. The activated sludge process can greatly affect the microbial structure and cause dilution of ARGs, MGEs, and ARB in activated sludge. Sedimentation processes cannot significantly affect the bacterial structure resulting from the selection of a secondary settling tank, so the relative abundance of ARGs, MGEs, and ARB in second clarifier effluent water was similar to activated sludge. The results highlighted the correlation of a comprehensive study of ARGs associated with MGEs and bacterial structure in the sewage treatment system, and this study might be technologically guided for activated-sludge design and operation to control antibiotic resistance.

## Data Availability

Not applicable.

## Supplementary Materials

The following supporting information can be downloaded at: https://www.mdpi.com/article/10.3390/ijerph20043593/s1, Table S1: Information about the four municipal sewage treatment plants (MSTPs) from which the water and sludge samples were collected; Table S2: PCR and quantitative re-al-time PCR primers of the 15 tetracycline resistance genes detected in this study; Table S3: Number of DNA sequences of ARGs carried by the corresponding genera revealed by searches in Antibiotic Resistance Database (Shadow means the significantly positive correlation between the genera and ARGs abundance); Figure S1: Occurrence patterns of 15 tet genes in influent water (IW), activated sludge (AS) and effluent water (EW) collected from Jiangxinzhou Municipal Sewage Treatment Plants (JXZ-MSTP) analyzed by electrophoresis of PCR products; Figure S2: Abundance of eight tetracycline resistance genes in influent water (IW), activated sludge (AS), and effluent water (EW) monthly sampled from Jiangxinzhou Municipal Sewage Treatment Plants (JXZ-MSTP); Figure S3: Neighbor-joining phylogenetic analysis of tetG diversity in in-fluent water (IW), activated sludge (AS) and effluent water (EW) collected from Jiangxinzhou Municipal Sewage Treatment Plants (JXZ-MSTP); Figure S4: Percentage of antibiotic resistance genes coding for resistance to different antibiotics in influent water (IW), activated sludge (AS), and effluent water (EW) in the four municipal sewage treatment plants; Figure S5: Relative abundance of different types of integrase genes in influent water (IW), activated sludge (AS) and effluent water (EW) sampled from the four municipal sewage treatment plants; Figure S6: Heatmap of the Pearson correlation coefficients between integrase genes and ARGs in influent water (IW), activated sludge (AS) and effluent water (EW) sampled from the four municipal sewage treatment plants (*n* = 12); Figure S7: Heatmap of the Pearson correlation coefficients between insertion sequences and ARGs in influent water (IW), activated sludge (AS), and effluent water (EW) sampling from the four municipal sewage treatment plants (*n* = 12); Figure S8: Relative abundance of insertion sequences (>1 × 10^−5^) in influent water (IW), activated sludge (AS) and effluent water (EW) sampled from the four municipal sew-age treatment plants. References [40,41,42] are cited in the Supplementary Materials.
