# Supplementary material for: Correlations among Antibiotic Resistance Genes, Mobile Genetic Elements and Microbial Communities in Municipal Sewage Treatment Plants Revealed by High-Throughput Sequencing
Source: Int J Environ Res Public Health. 2023 Feb 17;20(4):3593. doi: 10.3390/ijerph20043593 (PMC9965123; doi:10.3390/ijerph20043593)
Supplement: Supplementary file 1 [file ijerph-20-03593-s001.zip › ijerph-2168416-supplementary.pdf]

Correlations among antibiotic resistance genes, mobile genetic elements and microbial communities in municipal sewage treatment plants revealed by high-throughput sequencing

Authors: Fuzheng Zhao[1,2,3], Bo Wang[2], Kailong Huang[3], Jinbao Yin[3], Xuechang Ren[2], Zhu Wang[4]*, Xu-Xiang Zhang[3]*

Affiliation of all authors:

[1] Key laboratory of Yellow River Water Environment in Gansu Province, Lanzhou Jiaotong University, Lanzhou 730070, China;

[2] School of Environmental and Municipal Engineering, Lanzhou Jiaotong University, Lanzhou 730070, China;

[3] State Key Laboratory of Pollution Control and Resource Reuse, School of the Environment, Nanjing University, Nanjing 210023, China;

[4] Institute of Environmental Research at Greater Bay/Key Laboratory for Water Quality and Conservation of the Pearl River Delta, Ministry of Education, Guangzhou University, Guangzhou 510006, China

***Corresponding author**:

Xu-Xiang Zhang (Email: zhangxx@nju.edu.cn)

Zhu Wang (Email: wangzhu@gzhu.edu.cn)

Table S1. Information about the four municipal sewage treatment plants (MSTPs) from which the water and sludge samples were collected.

MSTP	JXZ	DC	WX	ZZ
**Location**	JiangXinZhou MSTP,Nanjing City, Jiangsu province China	DaChang MSTP,Nanjing City, Jiangsu province China	WuXi MSTP,WuXi City, Jiangsu province China	ZhengZhou MSTP, Zhengzhou City, Henan province China
**Latitude(N)**	32º0'56.10"	32º0'56.10"	32º0'56.10"	32º0'56.10"
**Longitude(E)**	118º41'2.95"	118º43'43.14"	120º19'50.60"	113º36'35.96"
**Capacity (t/day)**	640,000	100,000	100,000	100,000
**Biological treatment process**	Anoxic/oxic process	Three-Tank Oxidation Ditch	Anaerobic/anoxic /oxic and MMBR process	Oxidation Ditch
**Sample date**	5-Dec-11<br>9-Jan-12<br>19-Feb-12<br>26-Mar-12<br>28-Apr-12<br>28-May-12	26-Jul-12<br>2-Aug-12<br>17-Aug-12	10-Aug-13<br>13-Aug-13<br>16-Aug-13	Nov-12<br>Feb-13<br>May-13

Table S2. PCR and quantitative real-time PCR primers of the 15 tetracycline resistance genes detected in this study.

Gene		Primer sequence 5' --> 3'	Fragment size (bp)	Annealing temperature (°C)
*tet*A [a]	Forward	GCTACATCCTGCTTGCCTTC	210	55
	Reverse	CATAGATCGCCGTGAAGAGG		
*tet*B [a]	Forward	TTGGTTAGGGGCAAGTTTTG	659	56
	Reverse	GTAATGGGCCAATAACACCG		
*tet*C [a] •	Forward	CTTGAGAGCCTTCAACCCAG	418	55
	Reverse	ATGGTCGTCATCTACCTGCC		
*tet*D [a]	Forward	AAACCATTACGGCATTCTGC	787	56
	Reverse	GACCGGATACACCATCCATC		
tetE [a] •	Forward	AAACCACATCCTCCATACGC	278	57
	Reverse	AAATAGGCCACAACCGTCAG		
*tet*G [a] •	Forward	GCTCGGTGGTATCTCTGCTC	468	55.5
	Reverse	AGCAACAGAATCGGGAACAC		
*tet*K [a]	Forward	TCGATAGGAACAGCAGTA	169	55
	Reverse	CAGCAGATCCTACTCCTT		
*tet*L [a]	Forward	TCGTTAGCGTGCTGTCATTC	267	56
	Reverse	GTATCCCACCAATGTAGCCG		
*tet*M [a] •	Forward	GTGGACAAAGGTACAACGAG	406	55
	Reverse	CGGTAAAGTTCGTCACACAC		
*tet*O [a]	Forward	AACTTAGGCATTCTGGCTCAC	515	50.3
	Reverse	TCCCACTGTTCCATATCGTCA		
*tet*A(P) [a]	Forward	CTTGGATTGCGGAAGAAGAG	676	55
	Reverse	ATATGCCCATTTAACCACGC		
*tet*S [a]	Forward	CATAGACAAGCCGTTGACC	667	56.5
	Reverse	ATGTTTTTGGAACGCCAGAG		
*tet*X [a] •	Forward	CAATAATTGGTGGTGGACCC	468	55
	Reverse	TTCTTACCTTGGACATCCCG		
*tet*O [b] •	Forward	GTGCCATCCTTGAGGAAAAA	189	58
	Reverse	TGCTTTCATACTGCACTCCG		
*tet*Q [b] •	Forward	GCTCACATTGATGCAGGAAA	153	58
	Reverse	CGTAGAAGCCCGGACAGTAA		
*tet*W [c] •	Forward	GAGAGCCTGCTATATGCCAGC	168	64
	Reverse	GGGCGTATCCACAATGTTAAC		
16S rRNA [d] •	Forward	CCTACGGGAGGCAGCAG	174	55
	Reverse	AATCCGCGGCTGGCA		

Note: The primers used in this study were designed according to Ng et al. (2001) [1], Szczepanowski et al. (2009) [2], and Lee et al. (1993) [3]. López-Gutiérrez et al. (2004) [4]

•: This primer set was used for real-time qPCR.

[a]: Ng, L.-K.; Martin, I.; Alfa, M.; Mulvey, M. Multiplex PCR for the detection of tetracycline resistant genes. Mol. Cell. Probe. 2001, 15, 209–215.

[b]: Szczepanowski, R., Linke, B., Krahn, I., Gartemann, K.H., Gutzkow, T., Eichler, W., Puhler, A. and Schluter, A. . Detection of 140 clinically relevant antibiotic resistance genes in the plasmid metagenome of wastewater treatment plant bacteria showing reduced susceptibility to selected antibiotics. Microbiology 2009, 155, 2306–2319.
[c]: Lee, C.; Langlois, B. E.; Dawson, K. A. Detection of tetracycline resistance determinants in pig isolates from three herds with different histories of antimicrobial exposure. Appl. Environ. Microbiol. 1993, 59, 1467–1472.
[d]: López-Gutiérrez, J. C.; Henry, S.; Hallet, S.; Martin-Laurent, F.; Catroux, G.; Philippot, L. Quantification of a novel group of nitrate-reducing bacteria in the environment by real-time PCR. J. Microbiol. Methods 2004, 57 (3), 399-407.

Table S3. Number of DNA sequences of ARGs carried by the corresponding genera revealed by searches in Antibiotic Resistance Database (Shadow means the significantly positive correlation between the genera and ARGs abundance).

	*tet*Q	*tet*C	*ant*(2')-Ia	*erm*B	*aph*(33')-Ib	*aph*(6)-Id	*ere*A	*cat*B3	*aac*(6)-Ib	*acr*B	*tet*39	*tet*W	*ant*(3)-Ia	*sul*I	*mex*B	*mex*F	*tet*E	*tet*M	*bla*$_{GES}$	*bla*$_{VEB}$	*tet*36	*bla*$_{OXA10}$	*tet*X	*bac*A	*sul*II
Hydrogenophaga																									
Dechloromonas																									
Geobacter																									
Sulfurospirillum																									
Anaerosinus																									
Phascolarctobacterium																									
Pseudomonas			**1**		**11**	**8**	**1**	**7**	**4**					**23**	**10**	**22**			**9**	**7**		**16**	**1**	**31**	
Cloacibacillus																									
Megamonas																									
Prevotella	**2**																								
Bacteroides	**15**	**1**																			**1**		**2**		
Anaeroarcus																									
Faecalibacterium												**1**													
Paludibacter												**2**													
Acinetobacter		**1**				**3**		**10**	**1**		**1**			**11**				**1**	**1**	**4**		**4**			
Thiothrix																									
Acidovorax																								**2**	
Zoogloea																									
Comamonas																								**1**	
Arcobacter																									

Parabacteroides	**1**
Trichococcus	

Figure S1. Occurrence patterns of 15 *tet* genes in influent water (IW), activated sludge (AS) and effluent water (EW) collected from Jiangxinzhou Municipal Sewage Treatment Plants (JXZ-MSTP) analyzed by electrophoresis of PCR products. (IW: influent water; AS: activated sludge; EW: effluent water; NC: negative control using ddH_2O as PCR template; PC: positive control using recombinant plasmids carrying target genes as PCR template; Marker: DNA Marker).

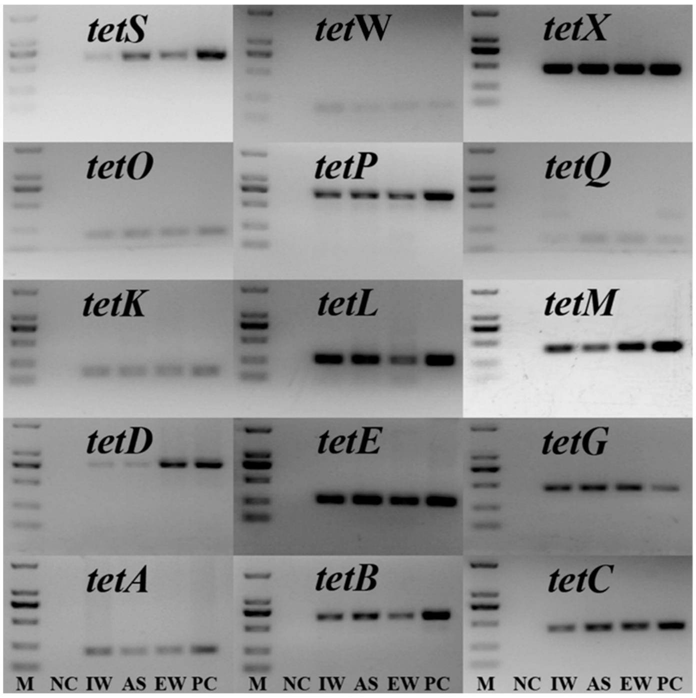

Figure S2. Abundance of eight tetracycline resistance genes in influent water (IW), activated sludge (AS), and effluent water (EW) monthly sampled from Jiangxinzhou Municipal Sewage Treatment Plants (JXZ-MSTP). The abundance was determined by quantitative real-time PCR and normalized to the abundance of 16S rRNA genes.

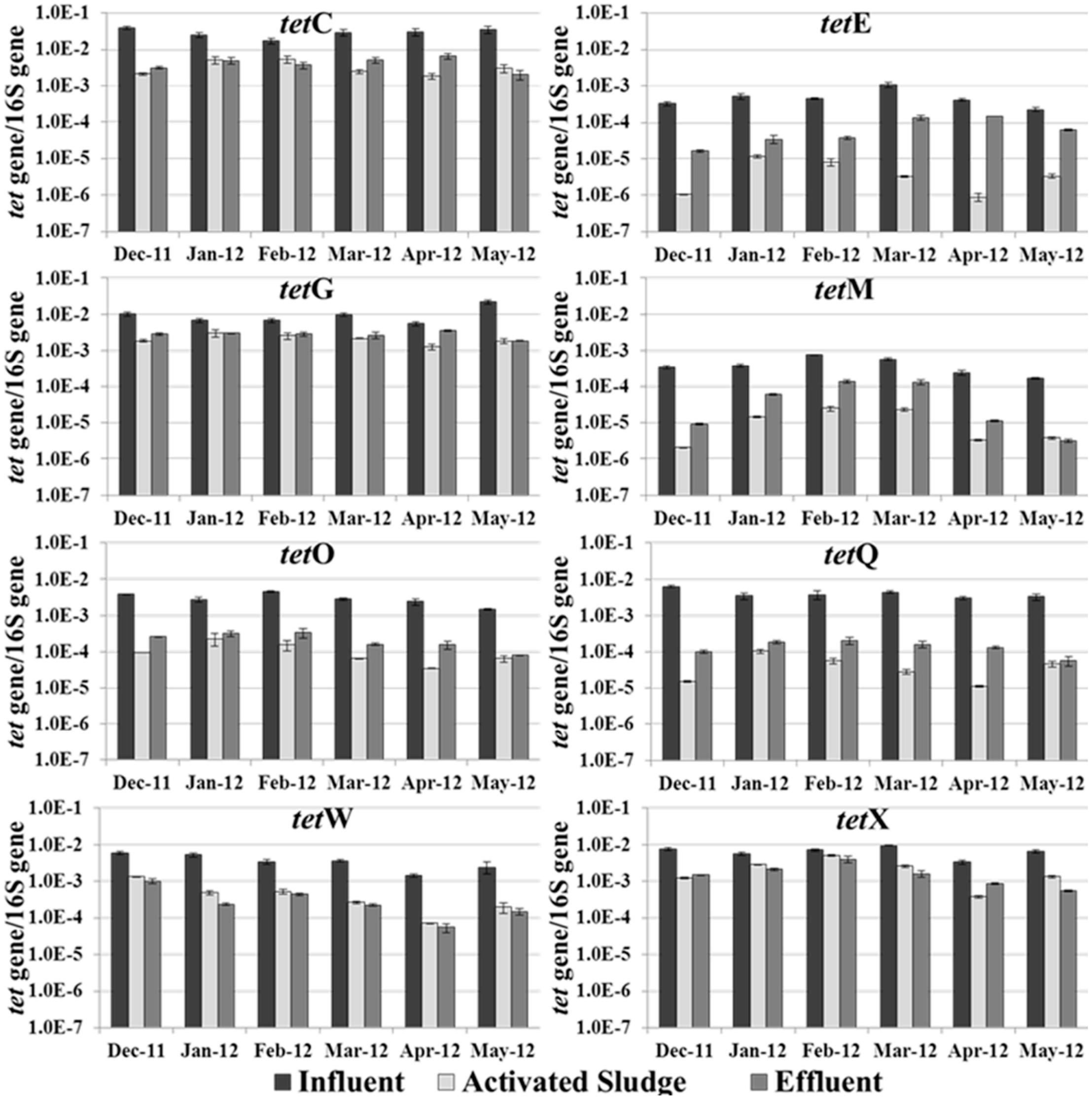

Figure S3. Neighbor-joining phylogenetic analysis of *tetG* diversity in influent water (IW), activated sludge (AS) and effluent water (EW) collected from Jiangxinzhou Municipal Sewage Treatment Plants (JXZ-MSTP). The tree was constructed using MEGA version 5 and bootstrap analysis with 1000 replicates was used to evaluate the significance of the nodes.

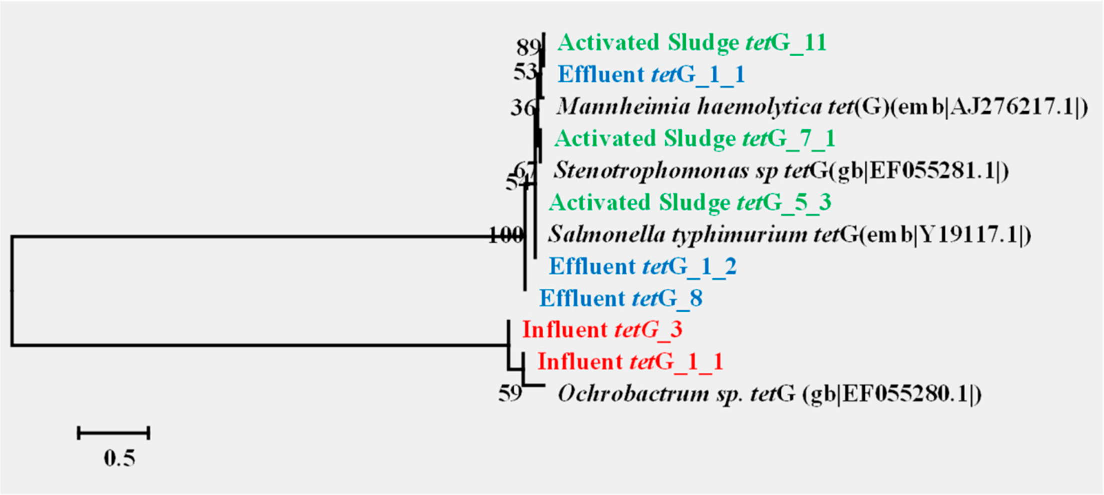

Figure S4. Percentage of antibiotic resistance genes coding for resistance to different antibiotics in influent water (IW), activated sludge (AS), and effluent water (EW) in the four municipal sewage treatment plants.

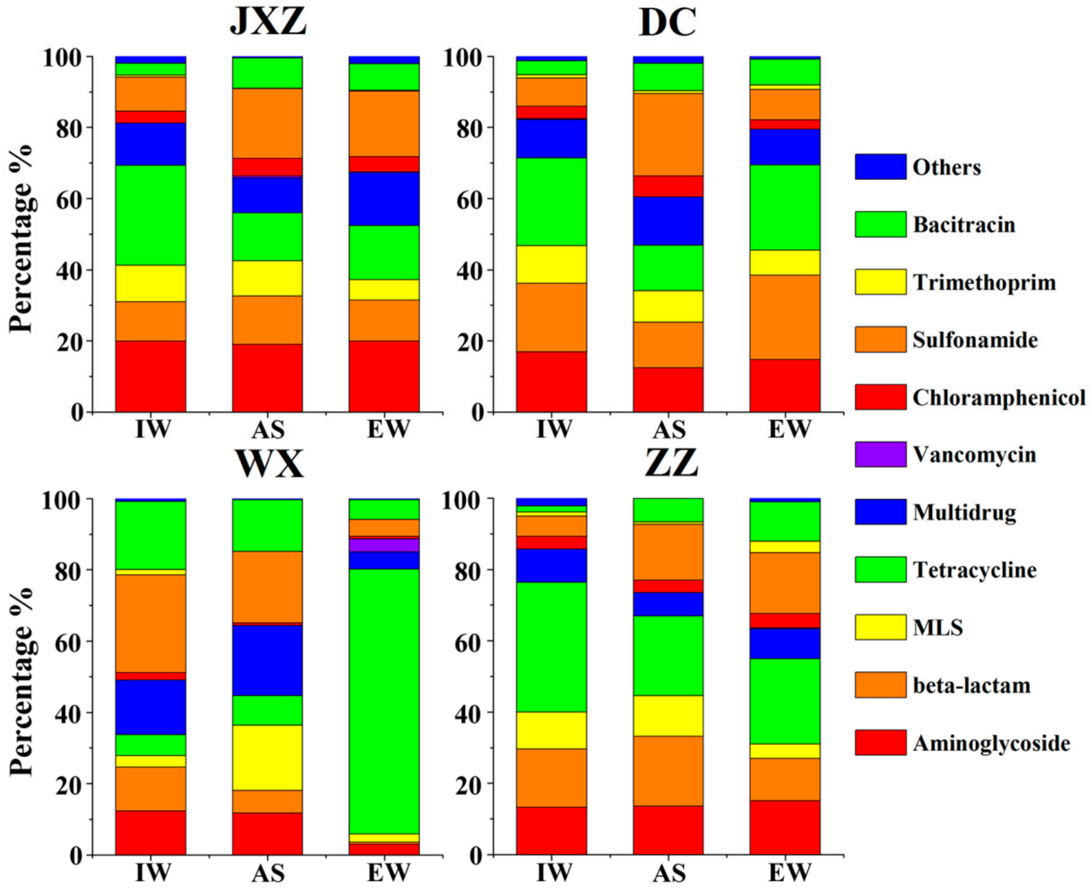

Figure S5. Relative abundance of different types of integrase genes in influent water (IW), activated sludge (AS) and effluent water (EW) sampled from the four municipal sewage treatment plants.

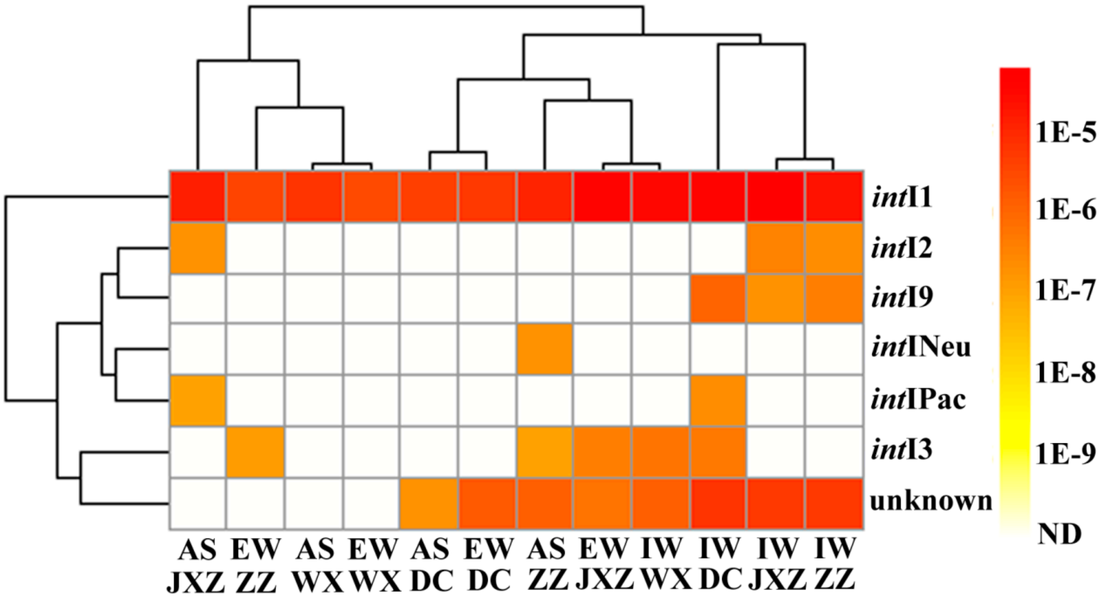

Figure S6. Heatmap of the Pearson correlation coefficients between integrase genes and ARGs in influent water (IW), activated sludge (AS) and effluent water (EW) sampled from the four municipal sewage treatment plants (n=12). The ARGs with abundance over 1.0E-05 abundance in any sample are shown. The Cluster Analysis (CA) is based on a distance matrix computed with Euclidean similarity of 25 ARGs and 7 integrase genes. The scale bar shows the variation range of the correlation coefficients. (*: R>0.5; P<0.05)

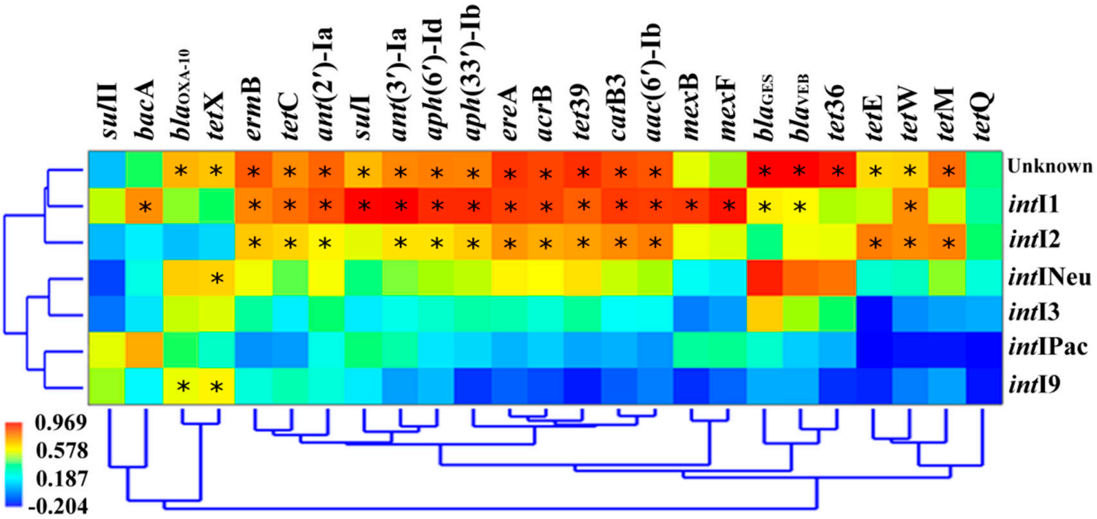

Figure S7. Heatmap of the Pearson correlation coefficients between insertion sequences and ARGs in influent water (IW), activated sludge (AS), and effluent water (EW) sampling from the four municipal sewage treatment plants (n=12). The ARGs or insertion sequences with relative abundance over 1.0E-05 in any sample are shown. The Cluster Analysis (CA) is based on a distance matrix computed with Euclidean similarity of 25 ARGs and 25 insertion sequences. Scale bar shows the variation range of the correlation coefficients. (*: R>0.5; P<0.05)

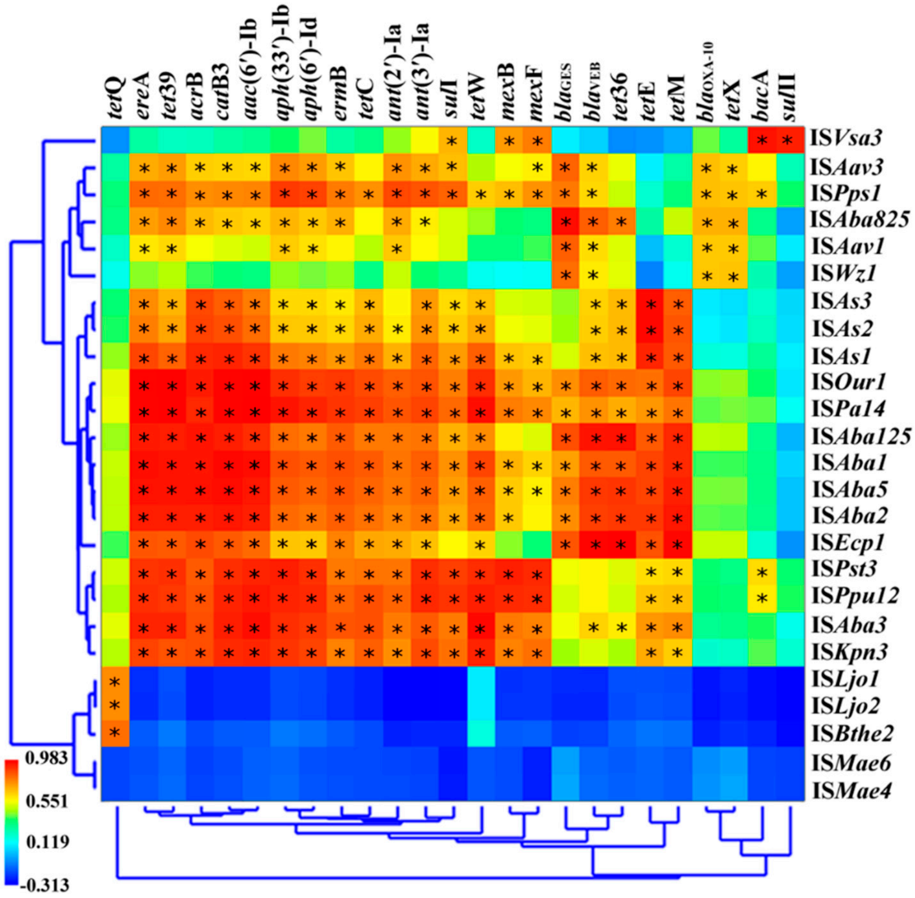

Figure S8. Relative abundance of insertion sequences (>1E-05) in influent water (IW), activated sludge (AS) and effluent water (EW) sampled from the four municipal sewage treatment plants.

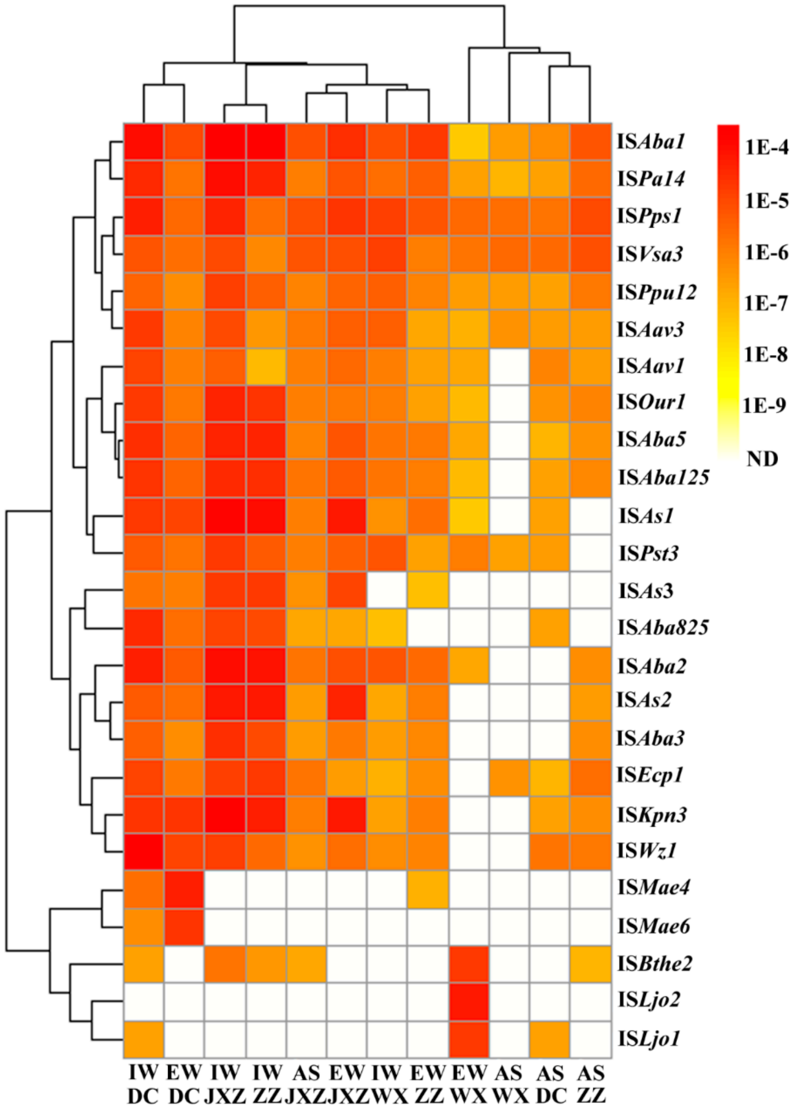

References

1. Ng, L.-K.; Martin, I.; Alfa, M.; Mulvey, M. Multiplex PCR for the Detection of Tetracycline Resistant Genes. *Mol. Cell. Probes* **2001**, *15*, 209–215, doi:10.1006/mcpr.2001.0363.
2. Szczepanowski, R.; Linke, B.; Krahn, I.; Gartemann, K.-H.; Gützkow, T.; Eichler, W.; Pühler, A.; Schlüter, A. Detection of 140 Clinically Relevant Antibiotic Resistance Genes in the Plasmid Metagenome of Wastewater Treatment Plant Bacteria Showing Reduced Susceptibility to Selected Antibiotics. *Microbiology* **2009**, *155*, 2306–2319, doi:10.1099/mic.0.028233-0.
3. Lee, C.; Langlois, B.E.; Dawson, K.A. Detection of Tetracycline Resistance Determinants in Pig Isolates from Three Herds with Different Histories of Antimicrobial Agent Exposure. *Appl. Environ. Microbiol.* **1993**, *59*, 1467–1472, doi:10.1128/aem.59.5.1467-1472.1993.
4. López-Gutiérrez, J.C.; Henry, S.; Hallet, S.; Martin-Laurent, F.; Catroux, G.; Philippot, L. Quantification of a Novel Group of Nitrate-Reducing Bacteria in the Environment by Real-Time PCR. *J. Microbiol. Methods* **2004**, *57*, 399–407, doi:10.1016/j.mimet.2004.02.009.
